# Supplementary material for: Hospital wastewater surveillance for SARS-CoV-2 identifies intra-hospital dynamics of viral transmission and evolution
Source: Appl Environ Microbiol. 2025 Jul 1;91(7):e00501-25. doi: 10.1128/aem.00501-25 (PMC12285251; doi:10.1128/aem.00501-25)
Supplement: Supplemental methods — Viral concentration and nucleic acid extraction from wastewater, and whole-genome sequencing of SARS-CoV-2 from clinical nasopharyngeal swabs. [file aem.00501-25-s0001.docx]

**Supplementary Methods**

**Hospital wastewater surveillance for SARS-CoV-2 identifies intra-hospital dynamics of viral transmission and evolution**

**Running Title:** Intra-hospital SARS-CoV-2 dynamics in wastewater

Medini K. Annavajhala^1+#^, Anne L. Kelley^1^, Lingsheng Wen^1^, Maya Tagliavia^1^, Sofia Z. Moscovitz^1^, Heekuk Park^1^, Simian Huang^1^, Jason E. Zucker^1^, Anne-Catrin Uhlemann^1#^

^1^Division of Infectious Diseases, Department of Medicine, Columbia University Irving Medical Center, New York, NY, 10032

+ Current affiliation and address:

Medini K. Annavajhala, Children’s Hospital of Philadelphia, Colket Translational Research Building, 3501 Civic Center Boulevard, Room 10010, Philadelphia, PA, 19104

# Corresponding authors:

Medini K. Annavajhala, PhD, [annavajham@chop.edu](mailto:annavajham@chop.edu)

Anne-Catrin Uhlemann, MD, PhD, [au2110@cumc.columbia.edu](mailto:au2110@cumc.columbia.edu)

**Keywords**: wastewater surveillance, SARS-CoV-2, COVID-19, hospital wastewater, wastewater-based epidemiology

**Viral concentration and nucleic acid extraction from wastewater:**

*Polyethylene glycol (PEG) precipitation:* Two aliquots of composite raw wastewater were concentrated from each site. 6.25 mL of glycine buffer (0.05M glycine, 3% beef extract, pH 9.6) was added to 43.75 mL raw wastewater for a total volume of 50 mL and centrifuged at 5,000-8,000 x g for 30 min at 4°C. The supernatant was removed using a serological pipette to avoid disturbing the pellet and passed through a 0.2 or 0.45 µm polyethersulfone (PES) filter. The filtrate was then incubated overnight with agitation at 4°C with 5X 1M PEG 8000 and 1.5M sodium chloride. The next day, the sample was centrifuged at 13,000 x g for 2 hours at 4°C. The supernatant was discarded, and the pellet was resuspended with 0.3 mL of 1X phosphate buffered saline (PBS) and stored at -80°C.

*InnovaPrep Concentrator Pipette*: Two 50 mL aliquots of composite raw wastewater from each site were centrifuged at 4,500 x g for 15 min at 4°C to remove debris. The supernatant was decanted into a fresh 50 mL tube, and 500 µL of 10% Tween20 was added. Samples were then filtered through an Innoveprep Concentrating Pipette Select using the ultrafiltration pipette tip (~0.01 µm pores) and 25 mM Tris FluidPrep Elution Fluid. One concentrated aliquot was stored without additional storage buffer, and the other was mixed with an equal volume of Zymo DNA/RNA shield for nucleic acid stabilization. Both aliquots were stored immediately at -80°C.

*Nucleic acid extraction:* For early samples from May 2021 – February 2022, RNA was extracted using the RNeasy Mini kit (Qiagen) with an input of 150 µL concentrated wastewater in 1:1 DNA/RNA Shield, and an elution volume of 60 µL. From January 2022 – March 2024, the Zymo DNA/RNA Miniprep Kit was used to isolate both DNA and RNA from 175 µL of wastewater concentrate in 1:1 DNA/RNA Shield and eluted in 75 µL RNA or 60 µL DNA. DNA was immediately stored at –20°C. The LunaScript RT Supermix Kit (NEB) was used to generate 30 µL single-stranded cDNA from 24 µL extracted RNA, which was stored at –20°C. Remaining RNA was aliquotted and stored at –80°C.

**Whole-genome sequencing of SARS-CoV-2 from clinical nasopharyngeal swabs:** Nasopharyngeal swabs from patients testing positive for SARS-CoV-2 were sequenced as previously described.^1^ Briefly, after undergoing SARS-CoV-2 testing by the NYP-CUIMC Clinical Microbiology laboratory, positive specimens were inactivated using an equal volume of Zymo DNA/RNA Shield and stored at –80°C by the Columbia University Biobank. Samples were then requested by our laboratory and underwent RNA extraction using the QIAmp 96 Viral RNA kit on a QiaCube HT (Qiagen) using the CDC-recommended protocol,^2^ with an input of 70 µL sample in 1:1 shield and elution volume of 100 µL. The LunaScript RT Supermix kit was used to generate cDNA, which then underwent tiled whole-genome amplification and library preparation using the ONT Midnight Expansion Kit and Rapid Barcoding Kit (RBK110.96, Oxford Nanopore). Sequencing libraries were pooled and sequenced on an Oxford Nanopore GridION with R9.4.1 flow cells. Genome alignment, consensus sequence generation, and lineage calling via Pangolin were performed via the Epi2Me wf-artic Nextflow pipeline (Oxford Nanopore).

**References**

1. Annavajhala, M. K., Mohri, H., Wang, P., Nair, M., Zucker, J. E., Sheng, Z., Gomez-Simmonds, A., Kelley, A. L., Tagliavia, M., Huang, Y., Bedford, T., Ho, D. D. & Uhlemann, A.-C. Emergence and expansion of SARS-CoV-2 B.1.526 after identification in New York. *Nature* **597,** 703–708 (2021).

2. *CDC 2019-Novel Coronavirus (2019-nCoV) Real-Time RT-PCR Diagnostic Panel*. (Centers for Disease Control and Prevention). at <https://www.fda.gov/media/134922/download>
