# Supplementary material for: Hospital wastewater surveillance for SARS-CoV-2 identifies intra-hospital dynamics of viral transmission and evolution
Source: Appl Environ Microbiol. 2025 Jul 1;91(7):e00501-25. doi: 10.1128/aem.00501-25 (PMC12285251; doi:10.1128/aem.00501-25)
Supplement: Supplementary file 2 [file aem.00501-25-s0002.pdf]

Supplementary Table S1. Weekly wastewater sampling frequency.

WASTEWATER SAMPLING SITE	START DATE	END DATE	TOTAL WEEKS OF SAMPLING	NUMBER OF SAMPLES COLLECTED PER WEEK	NUMBER OF WEEKS[1] (% OF TOTAL WEEKS)
**RB**	10/26/2021	2/21/2024	121	0	14 (12%)
				1	7 (6%)
				2	10 (8%)
				3	22 (18%)
				4	19 (16%)
				5	47 (39%)
				6	2 (2%)
**HQB**	6/9/2021	3/13/2024	144	0	10 (7%)
				1	7 (5%)
				2	15 (10%)
				3	35 (24%)
				4	23 (16%)
				5	52 (36%)
				6	2 (1%)
**HQA**	5/5/2021	3/13/2024	149	0	7 (5%)
				1	14 (9%)
				2	15 (10%)
				3	26 (17%)
				4	32 (22%)
				5	53 (36%)
				6	2 (1%)

[1] The number of weeks where *n* samples were collected throughout the sampling period are shown for each sampler.
