# Supplementary material for: Hospital wastewater surveillance for SARS-CoV-2 identifies intra-hospital dynamics of viral transmission and evolution
Source: Appl Environ Microbiol. 2025 Jul 1;91(7):e00501-25. doi: 10.1128/aem.00501-25 (PMC12285251; doi:10.1128/aem.00501-25)
Supplement: Supplementary file 3 [file aem.00501-25-s0003.pdf]

Supplementary Table S2. Lead-lag correlation analysis between wastewater viral concentration and confirmed clinical cases in HQA with shedding dynamics sensitivity analysis, with and without normalization by crAssphage concentration

CROSS-CORRELATION MODEL	**Wastewater Data Only[1]** t_0 (cases) ~ t_0 (WW) + t_{-7} (WW)				**Normalized Wastewater Data Only[2]** t_0 (cases) ~ t_0 (WW_{norm}^2) + t_{-7} (WW_{norm})			
ASSUMED VIRAL SHEDDING WINDOW[3]	**3d**	**7d**	**10d**	**14d**	**3d**	**7d**	**10d**	**14d**
HQA model R^2	0.33	0.30	0.30	0.33	0.32	0.29	0.29	0.32
HQA model *p*[4]	***	***	***	***	***	***	***	***
p (t_0 WW or WW_{NORM})	**	*	*	*	*	*	*	*
p (t_{-7} WW or WW_{NORM})	***	***	***	***	***	***	**	***

[1] Represents a subset of data from Table 1 with available concurrent SARS-CoV-2 and crAssphage viral concentration data (n=60 weeks; January 1, 2023 – March 1, 2024)

[2] WWnorm = wastewater viral concentration normalized by coincident crAssphage concentration using the formula:

Normalized wastewater viral load = wastewater viral load / - log(crassphage viral load)

[3] As calculated using hospital location data from patient electronic medical records; viral shedding window expressed in days after last confirmed clinical positive SARS-CoV-2 test

[4] p<0.05 (*); p<0.01 (**); p<0.001 (***)
