# Supplementary material for: Hospital wastewater surveillance for SARS-CoV-2 identifies intra-hospital dynamics of viral transmission and evolution
Source: Appl Environ Microbiol. 2025 Jul 1;91(7):e00501-25. doi: 10.1128/aem.00501-25 (PMC12285251; doi:10.1128/aem.00501-25)
Supplement: Supplementary file 4 [file aem.00501-25-s0004.pdf]

Supplementary Table S3. Lead-lag correlation analysis between wastewater viral concentration and confirmed clinical cases with sampling frequency sensitivity analysis

CROSS-CORRELATION MODEL	**Wastewater Data Only** t_0 **(cases)**[1] **~** t_0 **(WW) +** t_{-7} **(WW)**		
OBSERVATIONS PER WEEK (N)	**1**	**2**	**3**
HQA model R^2	0.01	0.01	0.13
HQA model *p*[2]	0.24	0.19	*
p (t_0 WW)	0.36	0.38	0.1
p (t_{-7} WW)	0.15	0.13	*
HQB model R^2	0.44	0.52	0.55
HQB model *p*[2]	***	***	***
p (t_0 WW)	***	***	***
p (t_{-7} WW)	***	***	***

[1] As calculated using hospital location data from patient electronic medical records; assumed viral shedding window of 14 days after first confirmed clinical positive SARS-CoV-2 test
[2] $p<0.05$ (*); $p<0.01$ (**); $p<0.001$ (***)
